# Micronutrients in Future Diets: Considerations for Dietary Iron and the Food Matrix Effects on Bioavailability

**DOI:** 10.1002/mnfr.70483

**Published:** 2026-04-24

**Authors:** Prachi Punetha, Tom F. O'Callaghan, Elaine K. McCarthy

**Affiliations:** ^1^ School of Food and Nutritional Sciences University College Cork Cork Ireland; ^2^ Co‐Centre for Sustainable Food Systems University College Cork Cork Ireland; ^3^ INFANT Research Centre University College Cork Cork Ireland

## Abstract

Adequate nutrition is an essential contributor to improved health, longevity, and quality of life in the population. The shift toward sustainable, plant‐based diets is driving trends for the development and adaptation of more plant‐based food ingredients in future diets. With this, nutrient bioavailability, particularly of iron, remains a critical consideration. Iron deficiency remains a global public health challenge, particularly amongst women, pregnant women, and young children. One major contributor to iron deficiency is inadequate dietary iron intakes, but there are other factors that hinder the body's ability to absorb and utilize iron effectively. Dietary iron comes in two forms: heme, found in animal products and absorbed more efficiently, and non‐heme, found in plant‐based foods and is less readily absorbed. The absorption of non‐heme iron is influenced by various dietary components, which can act as inhibitors or promoters. Optimising food and ingredient formulations accounting for the food matrix and potential impacts on iron bioavailability can support nutritional adequacy and warrants consideration to ensure future foods are both healthy and sustainable.

## Graphical abstract

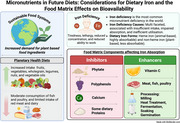

## 1 Introduction

The concept of a healthy and sustainable diet is not new, there has however been an increased emphasis on the adoption of healthy and environmentally conscious diets in recent years that put greater focus on the consumption of plant‐based foods, with reductions in animal‐based foods. Global food systems must adapt to provide nutritionally adequate diets for the population of approximately 8 billion people, with projections to approach 9.7 billion by 2064 [1]. To meet these needs while addressing environmental challenges such as rising greenhouse gas emissions and increased land use primarily linked to animal sourced food production [2], there has been a substantial shift towards sustainable food sources [2, 3]. The EAT‐Lancet Commission proposed the planetary health diet (PHD) rich in fruits, vegetables, wholegrains, legumes, nuts, and vegetable oils, with moderate consumption of fish and poultry, and limited intake of red meat and dairy [4], to encourage plant‐based eating and establish global targets for transforming food systems [5]. However, a complete shift toward plant‐based diets could put significant population groups at risk of inadequate micronutrient intakes [6].

More recently, the EAT–Lancet Commission (2025) emphasized the need for careful attention to iron, iodine, and calcium intakes within the PHD, particularly in populations with limited dietary diversity and among women of reproductive age due to their increased nutrient requirements related to menstruation, pregnancy, and lactation [7]. Beal et al. [8] conducted a modelling study using standardized dietary reference values (DRVs) and globally representative food composition data, while considering the elevated nutrient requirements of women of reproductive age, indicated that adopting the PHD could result in intakes of iron, calcium, and zinc falling below recommended levels. However, a recent randomized controlled trial in healthy adults found no difference in iron status between sustainable and healthy diets [9]. Although, the authors highlighted that the iron intake was predominantly non‐heme derived from plant sources, making the bioavailability highly dependent on the presence of co‐nutrients. Follow‐up sensitivity analysis, assuming moderate iron absorption and high phytate intake, indicated a high prevalence of inadequate iron intake, particularly among women of reproductive age [9]. The EAT‐Lancet Commission (2025) suggests that optimizing food choices within PHD reference ranges, such as increasing green leafy vegetables to improve iron intake, can support nutritional adequacy [7]; however, consideration of nutrient bioavailability remains critical when transitioning to predominantly plant‐based diets. For example, legumes, despite their substantial mineral content, exhibit poor bioavailability due to the presence of compounds such as phytates, potent inhibitors of iron and zinc absorption, and, in certain varieties, polyphenols that can bind to iron, thereby further impeding iron uptake [10].

Another concern in the shift toward sustainable diets from a mineral perspective is the growing emphasis on protein concentrates and isolates, driving increased use and incorporation of plant‐based protein ingredients. Focusing on protein isolation can overlook other aspects, as processing to extract and concentrate protein can also increase the concentration of compounds which reduce nutrient absorption and subsequent bioavailability [11]. Such compounds have been referred to as anti‐nutritional factors (ANF). D'Auria et al. [12] compared ANF levels in commercial plant ingredients and highlighted the importance of optimizing processing techniques to minimize ANFs while preserving protein, emphasizing that nutrient quality must be considered alongside sustainability in developing plant‐based foods. Consequently, with increasing adaptation or migration to plant‐based diets by some cohorts, increased understanding of nutrient content and its bioavailability in future food systems warrants greater attention to protect public health and prevent worsening crisis of mineral deficiencies. Iron is particularly critical in this context, as it is the most prevalent mineral deficiency worldwide, affecting an estimated 2 billion people and exerting considerable impacts on health and quality of life [13]. Therefore, this article provides a comprehensive review of dietary iron, iron deficiency and the key considerations for dietary iron bioavailability in the transition to more sustainable, healthy diets.

## 2 Iron Deficiency

Iron is a trace element that is vital for a range of biological functions, including energy metabolism, oxygen transport by red blood cells, cellular proliferation, enzymatic activities, and immune defense [14]. These functions become particularly critical during periods of increased demand, such as pregnancy and infancy, when hematopoiesis, growth, and development are at their peak [15]. The European Food Safety Authority (EFSA) has outlined Population Reference Intakes (PRI) across the lifespan for iron, as shown in Table 1.

**TABLE 1:** Population reference intake (PRI) value for iron from the European Food Safety Authority [16].

Age group	PRI (mg/day)
Infants (7–11 months)	11
Children (1–6 years)	7
Children (7–11 years)	11
Male (12–17 years)	11
Female (12–17 years)	13
Male > 18 years	11
Premenopausal female > 18 years	16
Postmenopausal female	11
Pregnancy	16
Lactation	16

Iron deficiency can significantly affect the well‐being of an individual, causing fatigue, sluggishness, and reduced work efficiency [17]. It is most common in pregnant women and young children, affecting 28%–85% of pregnant women [18] and over 50% of children aged 6–24 months across Europe [19, 20]. The deficiency is also prevalent among athletes, with rates ranging from 3%–11% in males and 15%–35% in females [21]. Since iron is crucial for haemoglobin synthesis, its depletion leads to microcytic hypochromic anaemia, a condition characterized by smaller‐than‐normal red blood cells with reduced haemoglobin content [22]. Anaemia is a major public health concern impacting one‐fourth of the global population [23], including approximately 500 million women of reproductive age. While anemia can arise from multiple causes, including micronutrient deficiencies (such as vitamin B12 and folate), chronic diseases, inherited red blood cell disorders, infections, and inflammation, iron deficiency remains the most prevalent nutritional cause of anemia worldwide [24]. The most recent data in the Global Health Observatory from the WHO suggests that 39.8% of children 6–59 months of age (in 2019), 30.5% of women of reproductive age, and 35.5% of pregnant women (both 2023 estimates) were anemic [25]. The World Health Organization has set a global target to reduce anemia in women of reproductive age by 50% by 2030, as part of its six core nutrition goals [25].

Iron deficiency is a complex global health issue, not solely caused by inadequate iron intake but also by factors that hinder the body's ability to absorb and utilize iron effectively. Absolute iron deficiency occurs when the body's iron stores are depleted, leaving insufficient iron to support bodily functions or replenish losses due to physiological or pathological factors [26]. This typically arises from inadequate dietary intake or absorption, increased iron demands during pregnancy and growth, and substantial losses due to menstruation and intestinal worm infections [27]. A major contributing factor is the limited intake of highly bioavailable heme iron from animal‐based foods, with plant‐based diets often providing less absorbable non‐heme iron [28]. Functional iron deficiency, on the other hand, occurs when inflammation (or infection) disrupts the body's ability to effectively utilize and absorb iron, even with adequate dietary intake [26].

## 3 Absorption of Iron

Iron from food is mainly absorbed by enterocyte cells in the duodenum and upper jejunum of the small intestine [29]. Iron absorption depends on its chemical form, with ferric (Fe^3^^+^) iron being poorly absorbed. Gastric acid facilitates the reduction of ferric (Fe^3^^+^) to ferrous (Fe^2^^+^) iron by the enzyme duodenal cytochrome B (Dcytb), enabling uptake via the divalent metal transporter 1 (DMT1) located on the apical membrane of enterocytes [30]. Within enterocytes, iron is either stored as ferritin, a process requiring the oxidation of ferrous iron to ferric iron by apoferritin [30], or exported into circulation through the basolateral membrane via ferroportin (FPN1), a transmembrane protein [31]. Ferroportin facilitates the transfer of ferrous iron from enterocytes into the bloodstream, where it is oxidized to ferric iron by the ferroxidase enzyme hephaestin, enabling its binding to plasma transferrin for systemic transport [32]. The process of iron absorption is essential for maintaining proper iron levels in the body since humans lack a mechanism for actively excreting iron [29]. Hepcidin is a liver‐derived 25 amino acid peptide hormone that regulates iron homeostasis by limiting intestinal absorption and restricting iron release from storage sites [32, 33]. When iron demand rises, hepcidin expression decreases, enhancing iron absorption and recycling to maintain an optimal supply [26]. Conversely, elevated iron levels or inflammation increase hepcidin expression, causing it to bind to the iron export protein ferroportin. This interaction triggers ferroportin internalization and degradation, reducing iron availability in circulation [33].

## 4 Bioavailability of Iron

Bioavailability refers to the fraction of a nutrient present in food, a diet, or a supplement, transported to specific tissues, and converted into its active form, thereby enabling its effective utilization in maintaining metabolic functions [34]. Consequently, the total iron content of a food is not an accurate measure of the amount of iron that is available for absorption and utilization [35]. Iron bioavailability is influenced by both physiological and dietary factors, including the iron status of the individual, factors such as obesity, infection, and inflammation, genetic disorders, and overall diet composition [36].

Dietary iron exists in two forms: heme iron and non‐heme iron [37]. Heme iron is present in the hemoglobin and myoglobin of animal‐based sources such as red meat, poultry, and fish, and is absorbed more efficiently than non‐heme iron. Non‐heme iron is found in plant‐based foods, as well as in dairy products, eggs, and the non‐heme fraction of animal tissues [34, 38]. While plant‐based foods often contain substantial amounts of iron, their absorption is limited by the chemical form of iron and the inhibitory nature of some compounds natively present in foods that interfere with iron uptake [39]. According to the Institute of Medicine (IOM), non‐heme iron absorption is estimated at approximately 16.8% based on a study on a self‐selected diet, whereas heme iron is conservatively assumed to be absorbed around 25% [40]. The WHO states that dietary iron absorption is largely influenced by the composition of the diet. As a result, the overall iron bioavailability in a whole diet ranges from 5% to 12% in individuals following vegetarian diets and increases to 14% to 18% in those who consume mixed diets [36].

In the diet, non‐heme iron is primarily present in its oxidized, ferric state, whereas heme iron typically exists in the reduced ferrous state, which is more readily transported into enterocytes [29]. Ferric iron tends to precipitate in solutions with a pH above 3, while ferrous iron remains soluble even at neutral pH levels. Consequently, ferric iron requires solubilization and chelation within the gastric environment to facilitate its absorption in the less acidic proximal region of the small intestine [41]. The solubilization and chelation of ferric iron are influenced by interactions with various dietary components in the intestinal lumen, which ultimately can modulate the absorption of non‐heme iron [42]. Based on their effects on iron bioavailability, these endogenous dietary constituents can be broadly categorized as either inhibitors or promotors of absorption [43]. Hence, the overall bioavailability of non‐heme iron from a diet largely depends on the interactions of these inhibitors and/or promotors present within that diet, which are summarized in Table 2 [44].

**TABLE 2:** Selection of dietary components affecting the absorption of iron.

Dietary component	Action	Mode of action	Reference
Phytate	Absorption inhibitor	Intact phytic acid can chelate iron, forming insoluble complexes, leading to its decreased bioavailability	[49]
Polyphenols	Absorption inhibitor	Polyphenols can form insoluble complexes with iron, thereby reducing its bioavailability	[58]
Calcium	Absorption inhibitor	Calcium can inhibit DMT1 transporter in a non‐competitive manner, resulting in reduced intestinal iron absorption Calcium salts form insoluble polymineral ligand complexes with iron and other dietary constituents, limiting their bioavailability	[34, 66]
Oxalic acid	Absorption inhibitor	Oxalates can bind to iron to form low solubility complexes, thereby reducing its bioavailability	[85, 86]
Proteins	Absorption inhibitor/promotor	Protein fractions influence iron solubility in the small intestine, thereby affecting its bioavailability	[78]
Organic acids (lactic acid, citric acid, tartaric acid)	Absorption promotor	Organic acids can chelate iron, leading to its increased solubility, thereby enhancing bioavailability	[91]
Organic acid (ascorbic acid)	Absorption promotor	Ascorbic acid can reduce ferric iron to a more bioavailable ferrous form. Ascorbic acid can enhance the solubility of both ferrous and ferric iron by forming soluble complexes Additionally, soluble complexes prevent its binding to inhibitors	[77] [93] [77]
Animal tissue	Absorption promotor	Provide highly bioavailable heme iron Enhance non‐heme iron absorption via the ‘meat factor’	[34, 104]

## 5 Inhibitors of Iron Absorption

### 5.1 Phytate

Phytate (myo‐inositol hexakisphosphate), which is naturally abundant in legumes such as mung beans, black beans, split beans, soybeans, and lentils, and thus prevalent in plant‐based diets is a well‐established inhibitor of non‐heme iron absorption, with the potential to reduce non‐heme iron uptake by 51%–82% [45, 46]. A study by Hurrell et al. [47] demonstrated that phytic acid concentrations as low as 4.9–8.4 mg/g in soy protein isolate strongly inhibited iron absorption, which increased four‐ to five‐fold when phytate content was reduced to below 0.01 mg/g, indicating that even small amounts can significantly impair absorption [47]. The human body lacks endophytases, the enzymes required for the breakdown of phytic acid, making it indigestible and unabsorbable in the small intestine [48]. Consequently, essential minerals such as iron, zinc, calcium, magnesium, and manganese become chelated by intact phytic acid, leading to reduced bioavailability [49, 50]. Among these minerals, the inhibitory effect of phytate on iron and zinc absorption is a major concern [50]. While its impact on zinc is less severe than on iron, improvements in iron absorption tend to enhance zinc absorption as well [51]. The removal or enzymatic degradation of phytate has been shown to significantly improve the absorption of these essential minerals [52, 53]. However, the extent of these improvements largely depends on the food matrix and processing methods, which modulate phytate content and its impact on mineral bioavailability.

The inhibitory effect of phytate is dose‐dependent, with reductions in iron uptake observed at phytate concentrations as low as 2–10 mg per meal [50]. The phytate‐to‐iron molar ratio serves as a useful indicator of its impact on absorption. Optimal absorption occurs when this ratio is below 1:1, with a preferable ratio under 0.4:1 in cereal or legume meals lacking absorption promoters. In mixed meals containing enhancers such as ascorbic acid (discussed later) or meat, the ratio should not exceed 6:1 to avoid significant inhibition of iron absorption [50, 54].

Phytate concentrations are generally higher in whole or minimally processed cereals compared to their refined counterparts [46]. Food processing and preparation techniques such as milling, heat treatment, fermentation, soaking, and germination can effectively reduce phytate content to varying degrees [36, 50]. These treatments promote dephosphorylation of phytic acid (myo‐inositol hexakisphosphoric acid) to lower myo‐inositol phosphate forms with a reduced inhibitory effect on mineral absorption [55], thereby improving iron bioavailability.

In addition to reducing phytate content, the broader food formulation and food matrix plays a critical role in determining iron bioavailability. Hurrell [50] reported that complete dephytinization in cereal‐ and legume‐based complementary foods increased iron absorption 12‐fold (from 0.99% to 11.54%) when the foods were reconstituted with water. However, this enhancement was negated when the same foods were reconstituted with milk, which inhibited iron absorption despite phytate degradation [50]. The inhibitory effect of milk on iron absorption is primarily attributed to its calcium content and casein, as discussed later in this review. Casein exerts this effect by binding iron through its phosphoserine clusters, thereby preventing its release in a free form available for intestinal absorption [56]. These findings suggest that strategies targeting phytate alone may be insufficient and that additional dietary factors influencing iron absorption must also be considered.

### 5.2 Polyphenols

Polyphenols, commonly present in plant‐based foods such as vegetables, legumes, cereals, fruits, spices, as well as beverages including red wine, cocoa, tea, and coffee, are well‐established inhibitors of iron bioavailability [57]. Their inhibitory effect is primarily attributed to their ability to chelate iron and form insoluble complexes, in a manner similar to phytate [38, 39, 58]. However, in a study on colored bean and sorghum varieties, which are rich in both phytate and polyphenols, it was demonstrated that while both compounds strongly inhibit iron absorption, the inhibitory effect of polyphenols was relatively smaller compared to that of phytate [14].

The phenolic compounds are released from the food matrix during digestion and bind iron within the intestinal lumen, thereby reducing its absorption [59]. Among polyphenols, tannins are high molecular weight compounds characterized by astringency and bitterness and are particularly potent inhibitors of iron absorption, forming insoluble iron complexes through hydrogen bonding mediated by hydroxyl and other functional groups [60].

Buerkli et al. [61] conducted a placebo‐controlled cross‐over study on European adults with hemochromatosis and reported a 40% reduction in iron absorption from an iron rich meal when consumed alongside a polyphenol supplement containing cocoa powder, black tea, and grape juice extract, thereby suggesting that iron absorption can be improved by avoiding concurrent consumption of iron rich meals and polyphenol rich foods. This inhibitory effect of polyphenol on iron absorption is dose‐dependent [59] and also varies with the specific type of polyphenol, as different compounds exhibit varying levels of impact on iron bioavailability [38]. A study in healthy young Thai women assessed the impact of turmeric and chili on iron absorption from a rice‐based meal. Despite turmeric containing higher amounts of polyphenols than chili, only chili was found to significantly reduce iron absorption, while turmeric showed no inhibitory effect [62]. Most dietary polyphenols, however, can significantly inhibit the absorption of non‐heme iron [59]. Kim et al. [63] used the Caco‐2 intestinal cell model to explore the impact of the dietary polyphenols epigallocatechin‐3‐gallate (EGCG) and grape seed extract (GSE) on transepithelial transport of iron. The study demonstrated that the inhibition of non‐heme iron absorption by polyphenols is attributed to the impaired iron transfer across the basolateral membrane of the enterocytes, likely due to the formation of non‐transportable polyphenol‐iron complexes. In another in vivo study on young women, Cercamondi et al. [64] examined the effect of polyphenols on more bioavailable forms of iron, such as chelated iron (eg., NaFeEDTA), in sorghum‐based meals. Iron absorption was higher from meals fortified with NaFeEDTA (4.6%) compared with those fortified with FeSO_4_ (2.7%), despite the presence of inhibitory polyphenols, highlighting the potential of chelated iron to mitigate polyphenol‐induced inhibition [64].

### 5.3 Calcium

Calcium is recognized as an inhibitor of iron absorption; however, unlike other inhibitors, it impacts the absorption of both non‐heme and heme iron [39]. Given that both heme and non‐heme iron absorption were equally affected, the inhibitory effect of calcium on iron absorption was initially thought to occur at a stage common to both forms, likely during the basolateral transport of iron from enterocytes to the plasma [65]. Studies have demonstrated that calcium inhibits intestinal iron absorption by acting as a noncompetitive inhibitor of the DMT1 transporter [66, 67]. However, evidence also suggests that calcium may exert its inhibitory action during the initial uptake of iron into the enterocytes [68].

Calcium is considered a relatively less potent inhibitor of iron absorption as compared to phytic acid and polyphenols [69], exhibiting dose‐dependent effects in simple meals, while showing limited inhibitory impact in complex meals that include absorption enhancers [14]. The dose‐dependent inhibitory effect of calcium on iron absorption was demonstrated in a study using a small bread‐based meal, where calcium intakes up to 300 mg led to a progressive reduction in iron absorption. A dose of 165 mg calcium resulted in approximately 50%–60% inhibition, regardless of whether the calcium was administered as calcium chloride or delivered through 150 mL of milk [70]. Overall, the extent to which calcium inhibits iron absorption depends on several factors, including the type of food, its natural calcium content, and the form of calcium added. For example, calcium salts can form insoluble polymineral ligand complexes with iron and other dietary constituents, limiting its absorption [34]. In a randomised controlled trial of iron‐deficient menstruating women, consuming an iron‐fortified milk product did not improve iron status, likely due to the presence of calcium and casein protein in the milk matrix [71]. With this in mind, cow's milk consumption has been highlighted as contributing to iron deficiency in young children through a number of mechanisms including its low native iron content, iron loss due to instances of occult intestinal bleeding associated with cow milk consumption during infancy, and inhibition of non‐heme iron absorption by calcium and casein, which are present in high concentrations [72]. In a recent systematic review of isotopically measured iron absorption in infants and children up to 2 years of age, researchers reported that although iron from breast milk has high bioavailability, cow's milk consumption reduces iron absorption and is associated with an increased incidence of iron deficiency and iron deficiency anaemia, making it an inappropriate primary nutritional source for infants less than 1 year of age [73, 74]. The differences in iron bioavailability between the two milk types can be attributed to the relatively lower calcium and casein content in human milk [75] presence of iron‐binding protein such as lactoferrin, ferritin, and transferrin [73]. Furthermore, studies suggest that the enhanced iron absorption from human milk involves more than its gross nutrient composition, with a milk matrix effect potentially responsible [76].

### 5.4 Protein

Dietary proteins affect iron bioavailability differently depending on their source [39]. Certain animal proteins, such as those from beef, chicken, and pork, have been shown to enhance iron absorption [77, 78]. In contrast, some other animal proteins including those derived from milk, eggs, and bovine albumin as well as plant proteins such as soy and wheat, generally act as inhibitors of iron absorption [34]. This has been reported in a radioiron absorption‐based study in healthy adults, in which replacing egg albumin with chicken protein or beef muscle protein fractions in a meal resulted in a significant increase in iron absorption [77].

The enhancing or inhibiting effects of different proteins on iron absorption may be attributed to the specific properties of their protein fractions, which influence iron solubility in the small intestine. For instance, proteins from pork improve iron solubility and absorption, while those from egg yolk reduce solubility, leading to lower iron uptake thus, variations in this solubility may be considered a key factor affecting the efficiency of iron absorption from different protein sources [78]. Even within the same food source, such as milk, proteins like casein and whey exhibit varying degrees of inhibition on iron absorption, with casein showing a stronger inhibitory effect. These differences result from the different digestion products of each protein, which can bind iron to form complexes. The strength and size of these complexes play a critical role in determining iron solubility and subsequent absorption [79]. In soybean, although phytate is considered the primary inhibitor of iron absorption, a study on non‐heme iron absorption from soy protein isolates demonstrated that even after complete phytate degradation, iron absorption was still approximately 50% lower compared to the egg‐white control, suggesting an inhibitory role of the soy protein itself [47]. Another study on soy protein isolates demonstrated that the use of enzyme‐hydrolyzed soy protein with minimal phytate content resulted in a 19‐fold increase in non‐heme iron absorption compared to the unmodified isolate. Although both major soy protein fractions, glycinin (11S) and conglycinin (7S), exhibited inhibitory effects on iron absorption, only the inhibition attributed to glycinin was alleviated through dephytinization, while conglycinin remained unaffected and continued to impair iron uptake [80]. However, a study on fermented soy sauces demonstrated a significant enhancement in iron absorption from rice meals. This enhancement is attributed not only to the absence of inhibitory soy proteins but also to the fermentation products beside organic acids [81].

### 5.5 Oxalic Acid

Oxalates are metabolic end products present in many plant tissues [82] and are generally classified as soluble oxalates, which are either free or bound to potassium and sodium, and insoluble oxalates, which are predominantly bound to calcium. The soluble form interferes with mineral uptake by binding to free mineral ions present in the small intestine, lowering their absorption [83]. Some leafy greens and root vegetables are particularly high in concentrations of both soluble and insoluble oxalates, such as spinach, amaranth, beetroot, and taro [82, 84]. High oxalate levels can lead to hyperoxaluria, increasing the risk of kidney stones and, in severe cases, renal complications [85].

Oxalates are considered undesirable due to their ability to bind with essential minerals, particularly divalent cations, at physiological pH to form complexes that have low solubility thereby reducing mineral bioavailability [85, 86]. The presence of oxalates and phytates in green leafy vegetables are primarily responsible for their poor iron bioavailability despite being rich in iron content [82]. However, the effect of oxalates on iron absorption depends on their chemical form in the food matrix. For instance, in spinach, which contains high levels of both oxalate and calcium, oxalate may exist predominantly as calcium oxalate. In this bound form, it has limited capacity to chelate ferric iron and therefore may exert only a minor inhibitory effect on iron absorption [87]. However, calcium oxalate has been shown to moderately reduce absorption under specific conditions. Adding 1 g of calcium oxalate to a cabbage‐based meal resulted in a 39% reduction in iron absorption [88]. Some studies suggest that oxalic acid has a limited effect on non‐heme iron absorption. One study compared non‐heme iron absorption from meals consisting of wheat bread rolls and either spinach (high oxalic acid content) or kale (low oxalic acid content), or kale with added potassium oxalate to match oxalic acid content of spinach. Iron absorption from kale alone (10.7%) and with added potassium oxalate (11.5%) showed no significant difference, while spinach led to 24% lower absorption. This suggested that the reduced absorption from spinach is likely due to its higher calcium and polyphenol content rather than oxalate [89]. Although oxalate has been shown to inhibit iron absorption, its most notable effect is on calcium absorption, primarily because calcium oxalate is poorly soluble [90].

### 5.6 Promotors of Iron Absorption

#### 5.6.1 Organic Acid

Organic acids such as ascorbic acid, lactic acid, citric acid, and tartaric acid enhance iron absorption by chelating iron thereby, increasing its solubility. Their ability to improve iron solubility depends on the iron source, food matrix, pH, ligand type, and processing conditions [91]. In a study of Indian women investigating the impact of various organic acids on non‐heme iron absorption from a rice‐based meal, geometric mean iron absorption increased by approximately 161% with the addition of 15 mg ascorbic acid; 204% with 1 g citric acid; 134% with 1 g tartaric acid; and 98% with 1 g L‐malic acid [88]. The pronounced improvement observed with a relatively small quantity of ascorbic acid underscores its role as the most effective promoter of non‐heme iron absorption. Ascorbic acid promotes absorption primarily by reducing ferric iron (Fe^3^^+^) to its more bioavailable ferrous form (Fe^2^^+^) [77]. Recent studies have demonstrated that one mole of ascorbic acid can reduce two moles of ferric iron within a pH range from 2.6 to 6.0. However, the efficiency of this reduction declines as pH increases, and ascorbic acid becomes ineffective as a reducing agent for Fe^3^^+^ above pH 6.0 [92]. Furthermore, ascorbic acid enhances the solubility of both Fe^2^^+^ and Fe^3^^+^ by forming soluble complexes [93], thereby preventing binding to inhibitors such as phytic acid or precipitation as ferric hydroxide [77], and ultimately facilitating efficient uptake by enterocytes. It has also been reported that ascorbic acid forms stable chelates with Fe^3^^+^ under the acidic conditions of the stomach [94], where the low pH limits the binding of inhibitory ligands [34] such as phytates [50] and tannins [64]. These chelates formed with ascorbic acid remain soluble at the more alkaline pH of the duodenum, facilitating absorption [94].

Ascorbic acid (AA) substantially enhances iron uptake even in the presence of absorption inhibitors such as phytates [95], polyphenols [44], calcium, and milk proteins [96]. However, since some of these inhibitors reduce absorption even at low levels, for example, 2 mg phytate can lower absorption by ∼18% [95], and 12 mg tannic acid by ∼30% [44], relatively high amounts of ascorbic acid are required to counteract their effects. Studies show that about 80 mg ascorbic acid is needed to overcome the effect of 25 mg phytate, while diets high in phytate (≥250 mg) may require several hundred milligrams of ascorbic acid (≥250 mg) [95]. Similarly, at least 50 mg of ascorbic acid is needed to counter the negative impact of a bread meal containing over 100 mg polyphenols [44]. For ascorbic acid to enhance iron absorption, it must be consumed together with the iron source. Studies have demonstrated that no increase in absorption occurs if ascorbic acid is taken several hours before the iron containing meal [97]. Hence, iron‐rich foods can be simultaneously consumed with ascorbic acid‐rich foods to enhance iron absorption. However, from a food technology perspective, incorporating ascorbic acid directly into iron‐rich food products presents technological challenges due to its instability during processing [92] and storage.

The effect of ascorbic acid on iron absorption is dose‐dependent however, the effect plateaus at higher ascorbic acid to iron molar ratios. For example, in a bread meal with 58 mg phytate, iron absorption doubled at a molar ratio of 1.6:1 (30 mg AA), increased by a further 40% at 2.7:1 (50 mg AA), and showed only an additional 8% improvement at 7.9:1 (150 mg AA) [44]. A more recent in vitro study by Sabatier et al. [98] reported that on increasing the molar ratio of ascorbic acid to iron from 2:1 to 4:1, only slightly enhanced iron absorption from ferrous sulfate, with no additional improvement in iron bioavailability from ferric pyrophosphate and iron‐casein complexes. Similarly, the addition of 60 mg of ascorbic acid at a 5:1 molar ratio to maize porridge fortified with highly bioavailable iron (NaFeEDTA) did not result in a significant improvement in iron absorption [99]. The optimal ascorbic acid to iron molar ratio for maximizing iron absorption varies with the inhibitor content of the meal. It has been demonstrated that meals with low to moderate levels of absorption inhibitors achieve a 50% or greater increase in iron uptake at molar ratios between 1:1 and 6:1 [100, 101]. However, meals with high levels of absorption inhibitors, such as soyabean‐based meals and cereal‐based meals may require substantially higher ratios of around 20:1 [102] and 40:1 [100], respectively, to significantly enhance absorption. A study by Beiseigel et al. [103] reported that, in the absence of ascorbic acid, women absorbed only around 2% of iron from maize and bean meals. Supplementing with ascorbic acid at a 15‐20:1 molar ratio (AA:iron) led to an approximately threefold increase in iron absorption across all meals. While most evidence on the enhancing effects of ascorbic acid comes from single‐meal studies, predicting the optimal ascorbic acid‐to‐iron combination for maximizing absorption in complete meals is more complex, due to variables such as total iron content, ascorbic acid‐to‐iron and ascorbic acid‐to‐inhibitor molar ratios, and the presence of other enhancers [92]. In the context of phytic acid, ascorbic acid supplementation emerges as an effective strategy to counteract the inhibitory effects of phytic acid and promote iron absorption [50, 95]. While for polyphenols, the addition of ascorbic acid to NaFeEDTA‐fortified meals substantially increased iron absorption to 13.6%, whereas reducing polyphenol content from 164 mg to 42 mg through laccase treatment produced only a marginal increase (4.6% to 4.8%) [64].

#### 5.6.2 Animal Tissue

Animal tissues enhance iron absorption by providing highly bioavailable heme iron and promoting the uptake of non‐heme iron from the meal [104]. Studies show that iron absorption from plant foods is generally below 5%, whereas absorption from animal sources such as fish, liver, and beef is significantly higher, ranging from 15% to 20%, largely due to the presence of heme iron [105]. However, beyond this, animal‐source foods have also been reported to enhance the absorption of non‐heme iron, further contributing to their superior bioavailability. This enhancing effect of animal tissue on non‐heme iron absorption is commonly referred to as the “meat factor” [34] and was first documented by Layrisse et al. [106], using in vivo studies. This study demonstrated a threefold increase in iron uptake from black beans when consumed with fish or veal. Similar findings were demonstrated in another study using the extrinsic tag method, showing a tenfold higher absorption of inorganic iron when ingested with veal muscle compared to maize [107]. The complete characterization of the meat factor remains limited and requires further investigation. Studies have shown that this factor varies among different animal protein sources. For example, Björn‐Rasmussen & Hallberg compared proteins with broadly similar amino acid compositions and demonstrated that beef muscle, chicken muscle, and fish increased iron absorption from a maize meal by two‐ to threefold, whereas egg albumin showed no significant effect [108]. Similar findings were reported in human absorption studies using liquid meals, where the inclusion of pork, beef, fish, or chicken resulted in approximately a two‐fold increase in non‐heme iron absorption compared with meals containing egg albumin [105]. Given the comparable amino acid profiles of these proteins, the differential effect on iron absorption is likely attributed to a factor present in muscle tissue but absent in egg white, or to compositional variations of digestion products such as peptones [108]. It has also been reported that consuming maize with beef digests containing cysteine‐rich peptides with intact thiol groups resulted in more than double the iron absorption compared to digests with oxidized thiol groups [109]. The promoting effect of cysteine has also been demonstrated in a study where cysteine improved the solubility of insoluble ferric iron, thereby increasing the uptake of both soluble and insoluble ferric iron by Caco‐2 cells. This study further indicated that glutathione must first be digested into cysteine or cysteinyl glycine to effectively promote iron absorption [110].

The effect of proteins on iron bioavailability could also stem from the iron binding ability of intact or partially digested proteins, and the formation of stable, soluble iron complexes with protein digestion products [111], with carboxyl groups identified as key binding sites [112]. Using a rat intestinal epithelial model, Horimoto et al. [113] demonstrated that pork proteins hydrolyzed by pepsin or microbial enzymes significantly enhanced the bioavailability of non‐heme iron, indicating a key role of peptide molecular weight in forming soluble iron‐peptide complexes. Notably, pepsin digestion in the presence of iron produced complexes that induced higher ferritin expression than iron added after hydrolysis, suggesting that iron–peptide complex formation during hydrolysis is more effective than simple iron–peptide mixtures. These findings highlight the potential of iron‐peptide complexes for food fortification and microencapsulation, offering improved bioavailability without compromising sensory qualities and minimizing gastrointestinal side effects associated with conventional iron supplements [113].

## 6 Conclusion

With the global population continuing to rise, healthy and sustainable diets, which focus largely on the transition to an increased consumption of plant‐based foods, are recommended to protect both human and planetary health. However, careful consideration of the nutritional implications of this transition, beyond the impact on macronutrient intakes, such as protein, is essential to safeguard public health and mitigate the risk of escalating the global burden of micronutrient deficiencies. Iron deficiency remains the most common micronutrient deficiency worldwide, with the impact of a transition to more plant‐based dietary patterns an important consideration for this critical nutrient. The bioavailability of iron in plant‐based foods is a concern, with dietary iron bioavailability influenced by multiple interacting factors, including the form of dietary iron, heme or non‐heme, as well as the presence of other native food components that can act as promotors or inhibitors of iron absorption. These concerns can be further exacerbated in the generation of plant‐based protein ingredients, whereby processing steps to extract and concentrate protein may result in an increase in compounds that inhibit iron absorption. Therefore, in the development of future plant‐based foods, protein ingredients and formulations, the entire food matrix and its subsequent effects on the bioavailability of critical micronutrients, like iron, need to be considered.

## Conflicts of Interest

The authors declare no conflicts of interest.

## Data Availability

Data sharing not applicable to this article as no datasets were generated or analysed during the current study.
